# Supplementary material for: Vision-based collective motion: A locust-inspired reductionist model
Source: PLoS Comput Biol. 2024 Jan 29;20(1):e1011796. doi: 10.1371/journal.pcbi.1011796 (PMC10852344; doi:10.1371/journal.pcbi.1011796)
Supplement: S1 Text — (PDF) [file pcbi.1011796.s001.pdf]

Vision-Based Collective Motion: A Locust-Inspired Reductionist Model - Supplementary Materials

David L. Krongauz[1*], Amir Ayali[2], Gal A. Kaminka[1]

[1] Computer Science Department, Bar-Ilan University, Israel
[2] School of Zoology and Sagol School of Neuroscience, Tel Aviv University, Israel
[*] Corresponding author: kingkrong@gmail.com

1 Measurement of Distance to Neighbors in Monocular, Non-stereoscopic Vision

In Fig A, the computation of distance from the subtended angle is shown for idealized circular-shaped agents. It can be seen that for circles, there exists a one-to-one relationship, given by $r = \frac{d}{2\sin 0.5\alpha}$ between the subtended angle and the distance. There is no ambiguity; hence the distance calculation is exact in the circular case.

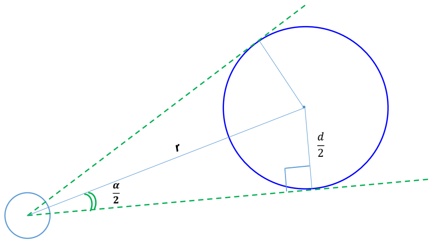

Figure A: **Exact distance r computation from subtended angle α for circular morphology.** Assumption of a circular (or spherical in 3D) shape of the agents, enables precise computation of distance r to a neighbor while employing the non-stereoscopic visual parameter of subtended angle α. The small circle on the lower left depicts the focal agent's visual sensor and the large circle on the right depicts a circular neighboring agent. Green lines represent the extreme rays toward the neighbor, as seen by the focal agent. The angle between the radius and the tangent extreme ray is always 90° by geometrical definition. Therefore, as shown $r = \frac{d}{2 \sin 0.5\alpha}$ where d is the circle's diameter and α the subtended angle measured.

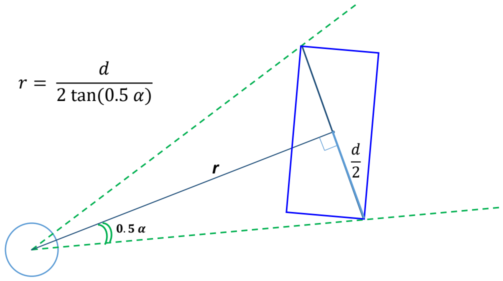

Figure B: **Approximate distance r computation from subtended angle α for elongated rectangles.** Assumption of rectangular shape for neighboring agents, enables only an approximate computation of distance r to the neighbor, using non-stereoscopic visual parameter of the subtended angle α. The small circle on the lower left depicts the visual sensor of the focal agent, and the large circle on the right depicts a circular neighboring agent. Green lines are the edge rays towards the neighbor, as seen from the focal agent. The angle between the edge rays is the observed subtended angle α. Therefore as shown in the figure $r = 0.5\frac{d}{\tan{(0.5\alpha)}}$

2 Derivation of Equation (7)

We know, from Eq. (4), that

$$r_j = \frac{1}{2}\|\boldsymbol{d}\| \cot\left(\frac{\alpha_j}{2}\right) = \frac{1}{2} d \cot\left(\frac{\alpha_j}{2}\right)$$

by differentiating Eq. (4) with respect to time t, we see (Eq. (6)) that

$$\boldsymbol{v}_{j,r} = \left(\frac{\partial}{\partial t} r_j\right) \hat{\boldsymbol{u}}_{j,r} = -\frac{1}{4} \frac{d\ \dot{\alpha}_j}{\sin^2(\frac{\alpha_j}{2})}\ \hat{\boldsymbol{u}}_{j,r}$$

where $\dot{\alpha}$ denotes the time derivative of the subtended angle.

Expressing $d = 2r_j \tan\left(\frac{\alpha_j}{2}\right)$ from Eq. (4) and substituting d into Eq. (6) results in the following derivation of the radial velocity $\boldsymbol{v}_{j,r}$,

$$\begin{aligned}
\boldsymbol{v}_{j,r} &= -\frac{1}{4} \frac{\dot{\alpha}_j\ d}{\sin^2(\frac{\alpha_j}{2})}\ \hat{\boldsymbol{u}}_{j,r} \\
&= -\frac{1}{4} \frac{\dot{\alpha}_j\ 2r_j\ \frac{\sin(\frac{\alpha_j}{2})}{\cos(\frac{\alpha_j}{2})}}{\sin^2(\frac{\alpha_j}{2})}\ \hat{\boldsymbol{u}}_{j,r} \\
&= -\frac{\dot{\alpha}_j}{2\sin\frac{\alpha_j}{2}\cos\frac{\alpha_j}{2}}\ r_j \hat{\boldsymbol{u}}_{j,r} \\
&= -\frac{\dot{\alpha}_j}{\sin\alpha_j} \boldsymbol{r}_j
\end{aligned}$$

which is what appears in Eq. (7).

3 Population Size N in the torus environment

We experimentally set the value of N in different arenas, to values that proved informative in the sense that they highlighted and clarified the differences between different strategies or other parameters. Below, we highlight the procedure and results used to set the values of N used in the torus arena. For other arenas, a similar experimental analysis was carried out.

We begin with the values that have already been set for the rest of the parameters: $R = 3$[BL], $\eta = 0.01$, length-to-width ratio set at 3. We then vary N. Figs Ca–Cb, show the results of 50 independent trials. For small N sizes, convergence to an ordered state is slow (at best) and probably non-existent. In the context of range-limited vision ($R = 3$[BL]), the sparse density undoubtedly inhibits overall convergence to ordered flocking. Increasing N leads to a higher long-term order parameter (Fig Cb), though clearly the rate of convergence differs (Fig Ca). Based on these results, we typically use population sizes $N = 60, 120, 180$ in the experiments in the torus arena.

Continuing from the aforementioned analysis, the choice of $N = 100$ for our principal experiments is further justified by the observed trends in Figs S3(a) and S3(b). These figures underscore a saturation effect in the order parameter beyond $N = 100$, where increasing the population size yields negligible improvements in collective behavior fidelity. This plateau suggests that $N = 100$ is a representative value for dense swarm simulations, providing a realistic portrayal of swarming dynamics without incurring disproportionate computational costs. Therefore, $N = 100$ was selected as it aligns with our dual objectives of accurately capturing the emergent properties of swarming behavior and maintaining computational feasibility within our simulation framework.

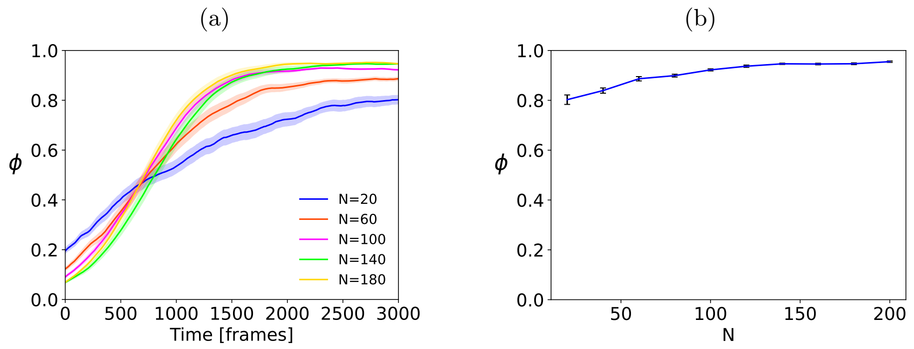

Figure C: **(a)** Time-dependent and **(b)** long-term sensitivity analysis for visual range N, in the torus arena. Means and standard errors are shown for 50 trials.

4 Influence of the Length-to-Width Ratio

We examine the influence of elongation on the emergence of order in flocking. In nature, nymph lengths vary as they grow to adulthood. A question also arises (when discussing the visual perception of the locust) regarding the inclusion of out-stretching legs in the perceived image. Based on our own measurements of the locust in our laboratory, we have chosen a body length-to-width ratio of 3 (i.e., length is three times the body width) as a baseline, which was used in the experiments reported above.

In addition, we sought to examine whether these settings influence the results. In particular, different models exist for vision-based flocking, for the case where agents are circular [51, 56, 58]. If the models introduced in this study are insensitive to the length-to-width ratio, then perhaps these other models could be just as useful in informing our understanding of how vision-based flocking may work in locusts (or other species that are elongated).

We, therefore, experimented with other ratios: a ratio of 1:1 (agents are perfect square), a 3:1 ratio, and a ratio of 6:1. Figs D and E report on the results from these experiments, in all environments (with the other parameters set as before $N = 100$, $R = 3$, $\eta = 0.01$, etc.). In all, we tested the principal model, as well as all three occlusion-handling strategies. As before, we conducted 50 independent trials in each setting and presented the means and standard errors.

Fig D shows the order parameter evolving over time in various settings. The subfigures are arranged by columns (different ratios) and rows (different arenas). The left column of the figures (Figs (a), (d), (g)) shows the results for ratio 1:1, the middle column (Figs (b), (e), (h)) show the results for the baseline ratio 3:1 used in the main group of experiments as reported above, and the rightmost column of figures (Figs (c), (f), (i)) show the results for ratio 6:1. The top row shows the results from the toroidal arena, the middle row shows results for the narrow corridor arena, and the bottom row shows the results for the narrow ring arena.

The figures show that the evolution of order, over time, is greatly influenced by the body length-to-width ratio. This is generally true for all the models; thus, we conclude that the body length ratio is an important factor in the convergence rate. Qualitatively, we note that higher ratios (elongated morphology) improve the rate of order increase in both the torus and corridor arenas. We believe these less-constrained arenas are closer to natural environments than the small ring-shaped arena (where greater elongation reduces—-possibly eliminates–the rate of order increases over time.

Fig E displays the long-term order parameter (ϕ at $t = 3000$) across different arenas and length-to-width ratios, offering a snapshot of Fig D at $t = 3000$. The torus arena shows more consistent model performance across various ratios, unlike other arenas. Notably, the COMPLID model generally excels in different settings, though this requires further study. As with Fig D, these findings underscore the importance of considering the length-to-width ratio in model evaluations for specific species.

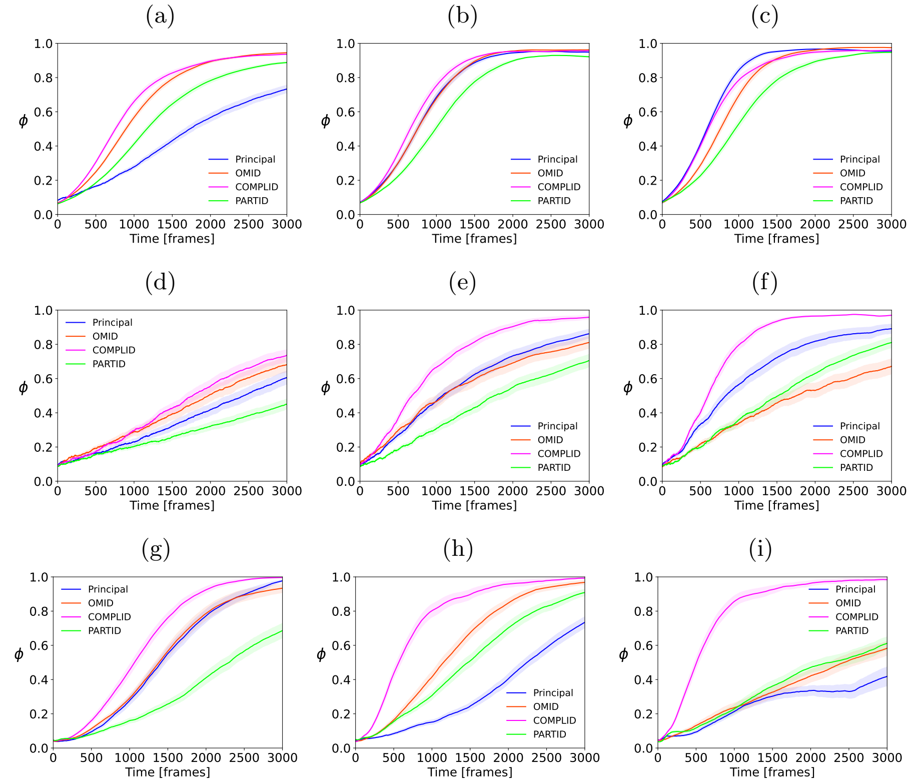

Figure D: Order measure ϕ evolving in time $t = 1 \ldots 3000$, for different body length-to-width ratios (left to right columns: 1:1, 3:1, 6:1) and arenas (top to bottom rows: torus, corridor, ring). The results are shown for the principal model, and the three occlusion-handling strategies. It is evident that the trend of order parameter evolution, for all models, depends significantly upon the length-to-width ratio.

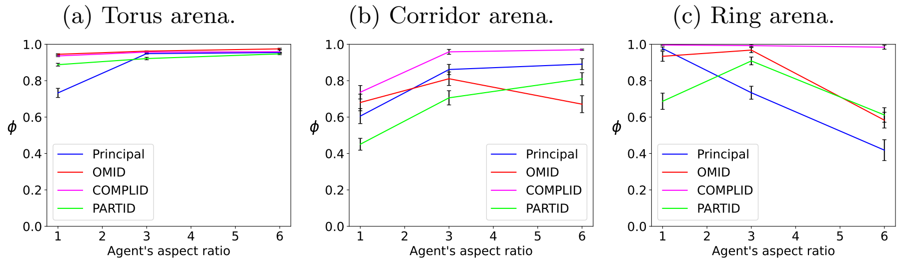

Figure E: Long term order (ϕ at $t = 3000$), for different body length-to-width ratios (horizontal axis in each subfigure), for different arenas (see captions). The results are shown for the principal model, and the three occlusion-handling strategies. Mean values and standard errors shown for 50 independent trials.
